# Management of clinically N0 neck in oropharyngeal carcinoma

**DOI:** 10.1007/s00405-019-05314-x

**Published:** 2019-02-07

**Authors:** Lauri Jouhi, Timo Atula, Antti Mäkitie, Harri Keski-Säntti

**Affiliations:** 1 Department of Otorhinolaryngology - Head and Neck Surgery, Helsinki University Hospital, University of Helsinki, P.O. Box 263, 00029 HUS Helsinki, Finland; 2 Division of Ear, Nose and Throat Diseases, Department of Clinical Sciences, Intervention and Technology, Karolinska Institutet and Karolinska Hospital, Stockholm, Sweden; 3 Research Program in Systems Oncology, Faculty of Medicine, University of Helsinki, Helsinki, Finland

**Keywords:** Head and neck cancer, Oropharynx, Carcinoma, HPV, p16, Regional recurrence

## Abstract

**Purpose:**

Only a minority of patients with oropharyngeal squamous cell carcinoma (OPSCC) are diagnosed without regional metastasis (cN0). Studies focusing on the management of cN0 neck in OPSCC are scarce.

**Methods:**

We reviewed all OPSCC patients treated at our institution with cN0 neck between 2000 and 2009. The treatment of neck and pattern of regional control was analyzed. Median follow-up was 5 years (range 3.5–9.0) or until death.

**Results:**

Of the total 313 OPSCC patients treated within the period, 56 (18%) presented with cN0 neck. Of them, 51 (91%) received completed treatment with curative intent: 46 (90%) underwent elective neck treatment with either neck dissection ± (chemo)radiotherapy (C)RT (*n* = 23) or (C)RT (*n* = 23). A regional recurrence occurred in three patients (6%) and they all had a p16-negative soft palate midline primary tumor. Two of these patients had received RT on the neck.

**Conclusions:**

While the overall prognosis of OPSCC is generally favorable and regional recurrences are infrequent, soft palate tumors, that are usually p16 negative, may form an subgroup warranting more aggressive treatment despite the clinical appearance of early stage.

## Introduction

One of the most current issues in head and neck oncology is the treatment of oropharyngeal squamous cell carcinoma (OPSCC) [1, 2]. The incidence of the HPV-related OPSCC is increasing [3]. It has been shown to have remarkably better prognosis compared with HPV non-related OPSCC, which is typically associated with long-term tobacco and alcohol consumption [4]. This has raised concern about possible overtreatment of HPV-related OPSCC and unnecessary treatment-related morbidity [5]. Ongoing studies are examining whether the treatment of patients diagnosed with HPV-related OPSCC could be de-escalated without compromising survival [1, 2].

Traditionally, treatment options for OPSCC include either primary surgery with or without postoperative (chemo)radiotherapy ([C]RT) or definitive (C)RT with surgery as a salvage option. These treatment options are considered to be equal in terms of survival outcome. However, the current opinions are derived from retrospective evaluations [6]. Currently, many prospective randomized trials on OPSCC management are ongoing, but still unfinished [2]. In many centers, definitive (C)RT has been favored in the treatment of tongue base tumors because of the high morbidity related to open surgical treatment. Recently, transoral robotic surgery has been developed to enable transoral removal of small (T1–T2) previously transorally inaccessible OPSCC tumors with low morbidity [7]. Further, the chosen treatment modality of the neck often corresponds to the treatment of the primary tumor.

Oropharyngeal cancer typically presents with a lump in the neck as the first symptom [8]. Especially, HPV-related OPSCC is often characterized by a small primary tumor with advanced neck disease. We have previously evaluated management of the neck with clinically detected regional lymph node metastasis (cN+) in OPSCC [9]. Only a minority of OPSCC patients present without clinically detected regional lymph node metastasis at the time of diagnosis (cN0) [10]. According to a common principle, elective neck treatment is indicated if the risk of occult metastasis is considered to be at least 15–20% [11]. Studies focusing on this subgroup of patients are infrequent. In this study, we retrospectively evaluated treatment approaches and outcome of cN0 OPSCC patients in a 10-year series at our institution. Special emphasis was placed on the occurrence of regional recurrences and on the factors associated with regional failures.

## Materials and methods

**Fig. 1:**
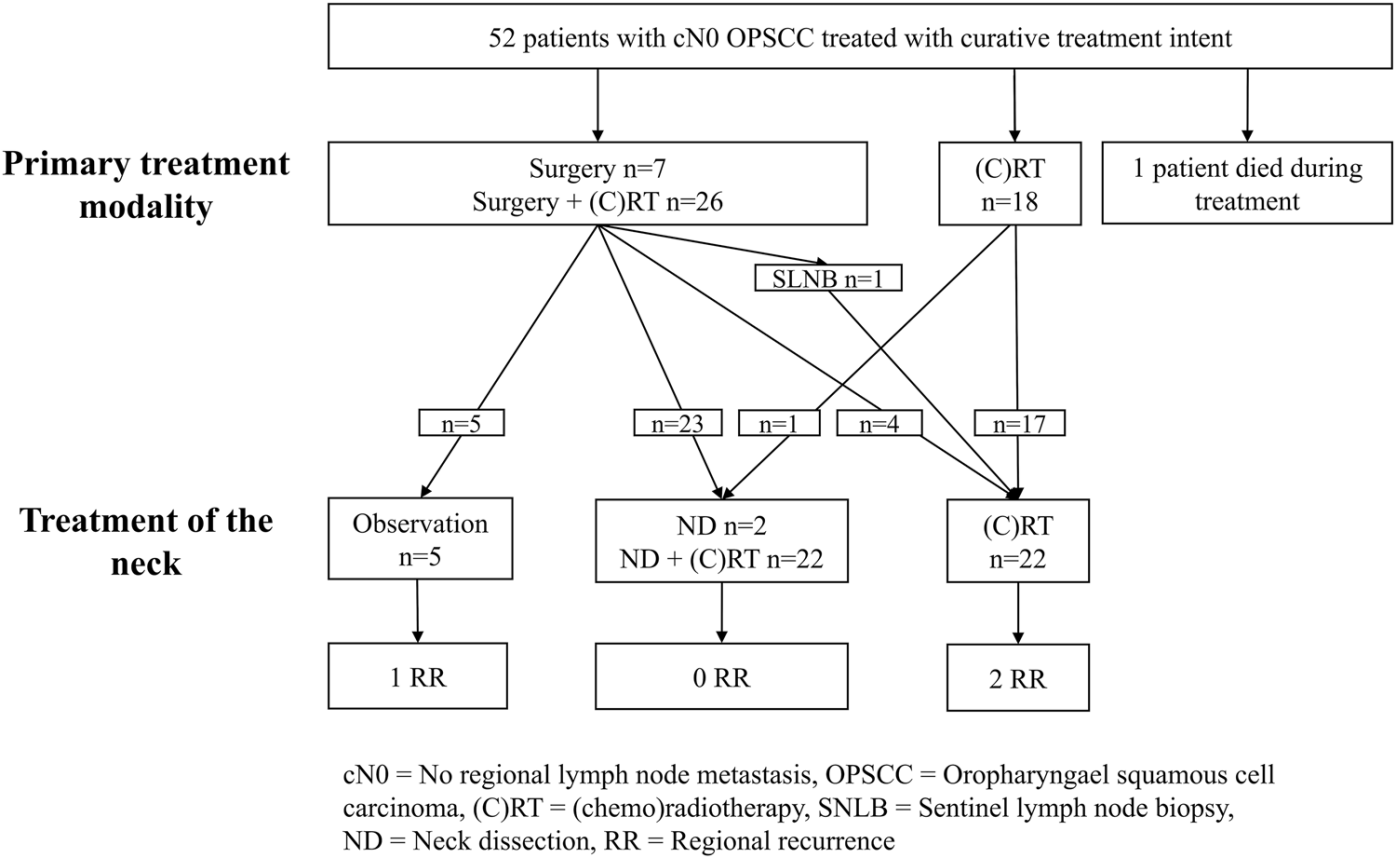
Flowchart of the patients with cN0 OPSCC

A total of 331 unselected consecutive patients were diagnosed with an oropharyngeal malignancy at the Helsinki University Hospital between 1st January 2000 and 31st December 2009 [9, 12]. From further analysis, we excluded those who had histology other than squamous cell carcinoma (*n* = 18), or had concurrent or earlier treatment for a head and neck malignancy (*n* = 16), or had a cN+ disease (*n* = 241). A total of 56 patients with a cN0 neck were included. Treatment with curative intent was initiated for 52 patients with a primary OPSCC and cN0 neck (Fig. 1). Clinicopathological data on patient and tumor characteristics, treatment, and follow-up were manually recorded from hospital regiestries. Dates and causes of death were provided by the Statistics Finland, and this data include fatal events caused by various cancer-related factors. Of the 52 patients, imaging methods prior to TNM assessment included magnetic resonance imaging of the neck in 44 patients, computed tomography in 32 patients and ultrasound in 6 patients. One patient underwent a sentinel lymph node biopsy (SLNB). Baseline clinicopathological data of the 52 patients with curative treatment intent are presented in Table 1. Institutional Research Ethics Committee of the Hospital District of Helsinki and Uusimaa approved the study design and a research permission was granted for the study. Tissue Microarray (TMA) method served in p16 expression evaluation from primary tumors as described elsewhere [13]. Median follow-up was 5 years (range 3.5–9.0) or until death.

**Table 1:** Baseline clinical characteristics of the 52 N0 OPSCC patients with curative treatment intent

		p16 positive	p16 negative	*p* value
No of patients^a^	52	20	24	
Sex				
Male	36	14	18	0.711
Female	16	6	6	
Smoking				
Never	4	3	0	0.003
Earlier	11	6	3	
Currently	28	6	18	
HPV				
Positive	15	14	1	< 0.001
Negative	24	5	19	
p16				
Positive	20			
Negative	24			
T class				
T1	8	2	5	1.000
T2	30	13	13	
T3	3	2	0	
T4a	11	3	6	
Site				
Lateral wall	23	14	5	0.001
Anterior wall	11	3	7	
Superior wall	16	2	12	
Posterior wall	2	1	0	

^a^In eight patients, p16 status was not available

*Lateral wall* tonsils and tonsillar pillars, *Anterior* wall base of tongue and vallecula, *Superior wall* soft palate and uvula

SPSS Version 21.0 (SPSS, Inc., Chicago, IL, USA) served in statistical analysis of the data. Kaplan–Meier estimate with the log-rank test was used to study the survival of patients over the time. The follow-up time in recurrence-free survival (RFS) and regional recurrence-free survival (RRFS) were defined as time between treatment completion and detection of recurrence in RFS and regional recurrence in RRFS or end of follow-up. Only detection of recurrence was defined as an event. The follow-up time in disease-specific survival (DSS) was defined as time between treatment completion and end of follow-up or death with disease, and in overall survival (OS) as time between treatment completion and end of follow-up or death of any cause. A Chi-square test was used to cross-tabulate categorical data. Asymptotic and exact *p* values were used when best suitable. A double-sided *p* value below 0.05 was considered statistically significant.

## Results

### Treatment of the primary tumors

Table 2 shows the treatment modalities for the primary tumors according to different T classes and p16 status. Altogether, single treatment modality was given to 10 patients (surgery alone 7 and RT alone 3), definitive CRT was delivered to 15 patients, and 26 underwent surgery followed by (C)RT. Salvage surgery due to residual primary tumor after (C)RT was performed in one case. One patient died during postoperative RT.

**Table 2:** Treatment modality of the 51 patients with a completed treatment for N0 oropharyngeal squamous cell carcinoma according to T class and p16 status

	Sx	Sx + RT	Sx + CRT	Definitive RT	Definitive CRT
T1	5	1	0	0	2
T2	2	16	1	3	8
T3	0	2	0	0	1
T4	0	3	3	0	4^a^
Total	7	22	4	3	15
p16+ (20)	1	9	3	1	6
p16− (23)	5	12	0	1	5

*Sx* surgery, *RT* radiotherapy, *CRT* chemoradiotherapy

^a^One patient underwent salvage surgery after CRT

### Treatment of the neck

Table 3 shows the given neck treatment according to T class. Five patients were followed up only. An elective neck dissection was carried out in 23 patients (20 ipsilateral and 3 bilateral) including levels I–V in 12 patients, levels I–IV in 7 patients, and levels I–III in 3 patients. Contralateral ND included levels I–III in two patients. The information on the extent of one bilateral ND was not available. One patient underwent a bilateral salvage ND (ipsilateral and contralateral levels I–V) after definitive CRT. Elective RT was given to 22 patients (15 of them had also concomitant chemotherapy due to a large primary tumor). One patient underwent SLNB, which revealed a metastasis and thus therapeutic RT was delivered. Of the 33 surgically treated patients, 26 (79%) received postoperative (C)RT, and they all had RT both to the primary tumor site and to the neck.

**Table 3:** Treatment to the neck of the 51 patients with completed treatment for cN0 disease according to T class

	Follow-up	ND	RT	ND + RT	RT + ND
T1	4	1	3	0	0
T2	1	1	15	13	0
T3	0	0	1	2	0
T4	0	0	3	6	1
Total	5	2	22	21	1

*ND* neck dissection, *RT* radiotherapy

### Occult neck disease

An occult regional metastasis was observed in altogether 7 (22.6%) out of the 31 patients who either underwent ND (in 5 out of 25 cases), or SLNB (in 1 case), or watchful observation without elective neck RT (in 1 out of the 5 cases 4 months after diagnosis). Of these 7 occult metastases, 2 developed in the 21 patients (2/21, 9.5%) with early local OPSCC (T1–T2) and 5 developed in the 10 patients (5/10, 50%) with advanced local OPSCC (T3–T4).

### Recurrences

Of the 51 patients with completed treatment, an isolated regional recurrence (no recurrence at the primary site) developed in three (5.9%) patients (Table 4). They all had had a T2N0 p16-negative midline soft palate primary tumor, that had been treated with surgery. None of them had had elective ND, but two had received RT to the neck. Three patients developed a local recurrence without regional involvement, and one patient experienced a distant recurrence without locoregional recurrence.

**Table 4:** The three patients with regional recurrent disease

Patient number	1	2	3
Age	69	55	49
Sex	Female	Male	Male
Site	Soft palate	Soft palate	Soft palate
Side	Bilateral	Bilateral	Bilateral
T class	T2	T2	T2
N class	N0 (pN1)	N0	N0
p16	Negative	Negative	Negative
HPV	Positive	Negative	Negative
Treatment	Surgery + RT	Surgery + RT	Surgery
Reconstruction	No	No	No
ND	No (bilateral SNLB)	No	No
Neck RT	Bilateral (56 Gy)	Bilateral (60 Gy)	No
RRFS time (mo)	7	16	3
Recurrence side	Contralateral	Ipsilateral	Bilateral
Treatment	Palliative RT	ND + RT	ND + RT
Status (mo)	DWD (11.0)	DWD (12.9)	DNED (5.7)

*RT* radiotherapy, *ND* neck dissection, *SLNB* sentinel lymph node biopsy, *Gy* gray, *mo* month, *DWD* died with disease, *DNED* died with no evidence of disease

### Survival

The 5-year RRFS for the whole population was 93.8%. The 5-year RRFS for the patients with elective neck treatment was 95.2%, and for the patients with neck follow-up only was 80.0% (*p* = 0.153). The 5-year DSS for patients with either elective neck treatment or neck follow-up only were 81.5% and 75.0% (*p* = 0.815) and the corresponding figures for OS were 71.4% and 40.0% (*p* = 0.126), respectively. The 5-year RFS, DSS and OS in the whole cohort were 83.8%, 79.3% and 66.3%, respectively. The 5-year DSS and OS for p16-positive and p16-negative cN0 OPSCC were 95.0% and 65.6% (*p* = 0.021) and 95.0% and 39.1% (*p* = 0.001), respectively.

## Discussion

We reviewed 52 consecutive OPSCC patients with cN0 disease over a 10-year period at our tertiary care hospital. In this series, surgery and (C)RT were both represented as elective treatment approaches for the neck. In only 13% of patients with curative treatment intent for early-stage disease, the neck was left untreated and was watchfully observed. The oropharynx has a rich lymphatic drainage, and OPSCC has a high propensity for metastatic spreading to regional lymph nodes. We have previously reported that at our institution during the same period 82% of all OPSCC patients had a cN+ neck [9], and in the present series, among those 18% without clinical lymph node metastasis, one-fourth (22.6%) had occult metastases. In addition, in this series, three patients, all with a soft palate tumor, suffered a regional recurrence. Thus, our results support the paradigm that in OPSCC, patients only rarely present with cN0 neck and most of them in this subgroup still seem to need treatment to the neck due to the high-risk occult metastases [14].

In terms of disease control, elective (C)RT and ND are generally regarded as equal treatment methods [14]. However, for most surgically treated patients (79%), adjuvant treatment was offered because of the size of the primary tumor, occult histologically verified neck metastases, or other adverse histopathological findings.

A single treatment modality with either definitive RT or surgery for early-stage OPSCC is generally recommended [6, 15, 16]. Roden et al. [17] reviewed 3247 early-stage tonsil cancer patients, and reported that surgery followed by RT resulted in a significantly improved OS (81%) as compared with surgery (67%) or RT (63%) alone. One can assume that the multimodality treatment group included patients with metastatic lymph nodes or other adverse pathological features, and, therefore, the differences in survival seem considerable. The worst survival (52%) was among the group of surgically treated patients who did not have any elective treatment of the neck. Elective neck dissection provides important information regarding metastatic spread to regional lymph nodes necessitating postoperative RT or even CRT and thus the highest risk patients receive multimodality treatment [18]. This may explain the better outcome among surgically treated patients in their series [17]. Even though a single treatment modality is favored in early-stage disease, surgery alone seems to remain relatively rarely an option in OPSCC [17], as observed also in our series.

Treatment of OPSCC has been changing towards to a more oncological approach over the last few decades, as the rate of surgeries has been decreasing and patients receive more often CRT [19, 20]. However, according to some studies, patients with HPV-negative OPSCC are suggested to benefit from surgery [21, 22]. Our previous study revealed that surgery and oncological treatment were equally represented in Finland during 2000–2009. p16 status was not available at the time of treatment decision, and the chosen treatment did not differ according to p16 status. However, patients with anterior-wall OPSCC received more often oncological treatment than surgery [23].

Soft palate tumors are presumably more often diagnosed with cN0 neck compared with other oropharyngeal sublocalizations, such as tonsil or base of tongue [24]. During years 2000–2009 in Finland, 64% of the soft palate were OPSCCs which were diagnosed without nodal involvement, as in the lateral and anterior wall corresponding 19% and 22% (Jouhi L, unpublished data based on study data series, Oct. 24, 2018). High probability for soft palate tumors to be diagnosed without regional lymph node metastasis is probably related to HPV status. It is well established that soft palate OPSCCs are typically HPV negative [25], and that HPV-negative tumors less frequently present with regional lymph node metastasis [5]. Hence, in our material, regional recurrences did not occur in patients carrying a p16-positive tumor. However, in our series one of the patients, who developed a regional recurrence, had a p16-negative but HPV-positive tumor. It has been previously reported that patients with this kind of discordant pattern of HPV and p16 may have a poor prognosis inspite of HPV positivity [26].

SLNB has been shown to detect occult neck disease reliably in oral cancer and it can be used instead of elective neck dissection to stage the neck [27, 28]. Many studies on sentinel lymph node biopsy include patients with OPSCC as the examination can be technically performed in some OPSCCs [29]. The number of OPSCC patients in these studies, however, is usually low and the role of SLNB is less clear in OPSCC [29]. Presumably, SLNB is not often used in OPSCC. In our series, SLNB was performed only in one patient with a tumor in the soft palate, and it revealed an occult neck disease. The patient was thereafter given bilateral neck RT but despite the treatment, a regional recurrence developed contralaterally. Thus, even a complementary ND would not have saved this patient from recurrent disease.

It seems that surgery may gain popularity in early-stage OPSCC management [2]. Trans-oral robotic surgery may be a feasible minimally invasive strategy for primary tumor resection with good functional outcome [7, 30]. In addition, histopathological data derived from the primary tumor and ND specimens may enable treatment intensification for the high-risk patients, while patients without adverse features may be treated safely with surgery only [15]. With this strategy, the long-term side effects of (C)RT could be avoided. In addition, as second primary tumors are relatively common in OPSCC patients, an option remains to later deliver treatment with RT.

OPSCC without regional lymph node metastasis is a minor but challenging patient group. Although our investigation covered a 10-year consecutive OPSCC population from an area with 1.6M inhabitants, the patient series with N0 neck remained fairly limited. Small number of patients and low incidence of RR hindered further statistical analysis, and consequently robust conclusions from the results cannot be drawn. In spite of that, our material is a representative unselected cohort, as treatment of all head and neck cancers, including also early-stage disease, is centralized to our hospital within our catchment area. However, during the long inclusion period of this study, the treatment paradigm was changing towards a more definitive (C)RT-oriented approach, which resulted in some heterogeneity in the treatment.

We conclude that in our series regional recurrences were infrequent in patients treated for OPSCC with cN0 neck and occurred only in patients with soft palate tumors. In addition, the midline localization of the tumor may have had an impact on the outcome of these cases in our series. Therefore, this additional aspect may warrant careful consideration in the treatment planning. Our results suggest that while the current overall prognosis of OPSCC nowadays is favorable and regional recurrences are infrequent, soft palate tumors may form a more aggressive subgroup being usually p16 negative and requiring often more intensive treatment.
